# Treatment of Leptomeningeal Carcinomatosis Following Treatment of Cerebellar Metastasis of HER2+ (Human Epidermal Growth Factor Receptor 2 Positive) Breast Cancer: Case Report and Review of Literature

**DOI:** 10.7759/cureus.24008

**Published:** 2022-04-10

**Authors:** Parker D Smith, Lokeshwar S Bhenderu, Sarayu Kommuri, Erin E Fleener, Jason M Hoover

**Affiliations:** 1 Molecular and Cellular Medicine, Texas A&M (Agricultural and Mechanical College) Health Science Center, Bryan, USA; 2 Neurological Surgery, Texas A&M (Agricultural and Mechanical College) Health Science Center, Bryan, USA; 3 Neurological Surgery, Texas Brain and Spine Institute, Bryan, USA; 4 Oncology, Cancer Clinic, Bryan, USA

**Keywords:** leptomeningeal carcinomatosis, her2, intrathecal trastuzumab, radiosurgery, radiation necrosis

## Abstract

Leptomeningeal carcinomatosis (LC) after metastasis of breast cancer is a rare occurrence with potentially devastating complications. Treatment options are limited, and there is a lack of literature on this topic. We report the case of a 38-year-old woman with estrogen/progesterone receptor negative (ER/PR-), human epidermal growth factor receptor 2 positive (HER2+) invasive ductal carcinoma of the left breast who underwent bilateral mastectomies with axillary lymph node dissection and chemotherapy treatment. The patient returned 11 months later with persistent headaches. Imaging and resection found cerebellar metastasis of the breast carcinoma. The brain metastasis was treated with further chemotherapy and stereotactic radiosurgery. Follow-up imaging showed the development of small lesions outside the radiation site. Metabolic studies were performed to determine if the new lesions were due to tumor recurrence or radiation necrosis, but the studies were inconclusive as to the etiology of these lesions. The patient later developed LC that was successfully treated with full resolution of the disease using intrathecal trastuzumab. There are currently no consensuses on treatment guidelines for treating LC. Here, we demonstrate successful treatment of LC from an ER/PR-, HER2+ breast carcinoma with intrathecal trastuzumab.

## Introduction

Only 20-30% of all invasive breast cancer is estrogen/progesterone receptor negative (ER/PR-), human epidermal growth factor receptor 2 positive (HER2+); approximately 30% of these tumors later develop brain metastases [1,2]. The advent of HER2 targeting therapeutics, such as trastuzumab and pertuzumab, made susceptible breast cancers much more treatable. In addition, smaller breast-to-brain metastases can clinically be treated effectively with radiation therapy. The risk of radiation necrosis following radiation therapy to the brain is approximately 26% and can result in seizures, mass lesions, or focal deficits [3].

Leptomeningeal carcinomatosis (LC) is a rare occurrence, but 33% of patients with previous brain metastasis are reported to ultimately develop LC [4]. LC is the spread of malignant cells to the leptomeninges by way of the vascular system or direct flow of cerebrospinal fluid from previous lesions. Treatment options are limited and occasionally ineffective due to the severity of the disease. The standard of care for LC has been radiation therapy or chemotherapy, but intrathecal trastuzumab has proven to be successful in recent studies of patients with HER2+ breast cancer [5].

## Case presentation

**Figure 1:**
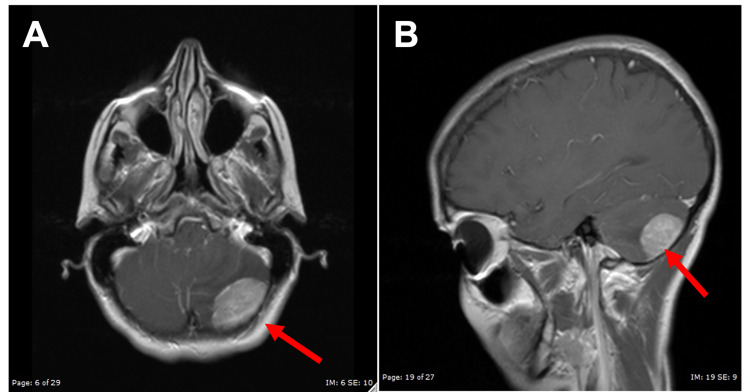
MR Images at Presentation of Headaches (A) Axial T2-weighted MR images showing cerebellar metastasis at the left posterior fossa (red arrow). (B) Sagittal T1-weighted MR images with gadolinium contrast showing cerebellar metastasis at the left posterior fossa (red arrow).

A 38-year-old woman with no previous medical history presented with a palpable mass in her left breast. On biopsy, the mass was found to be estrogen and progesterone receptor negative (ER/PR-), human epidermal growth factor receptor positive (HER2+) grade three invasive ductal carcinoma. Fine-needle aspiration and positron emission tomography (PET) showed disease in her ipsilateral axillary lymph nodes. She was initiated on a standard of care treatment at the time with neoadjuvant dose-dense doxorubicin and cyclophosphamide with weekly paclitaxel and trastuzumab. After the completion of paclitaxel, bilateral mastectomies and axillary lymph node dissection with breast reconstruction were performed and confirmed ER/PR-, HER2+ carcinoma in her left breast. She received adjuvant radiation to her left supraclavicular region, left internal mammary lymph nodes, and bilaterally to the chest wall. She completed one year of trastuzumab. Eleven months after completing trastuzumab, she presented with a week of persistent headaches. Brain magnetic resonance imaging (MRI) found a 4 x 2.3 x 2 cm mass at the left posterior fossa (Figures 1A, 1B).

**Figure 2:**
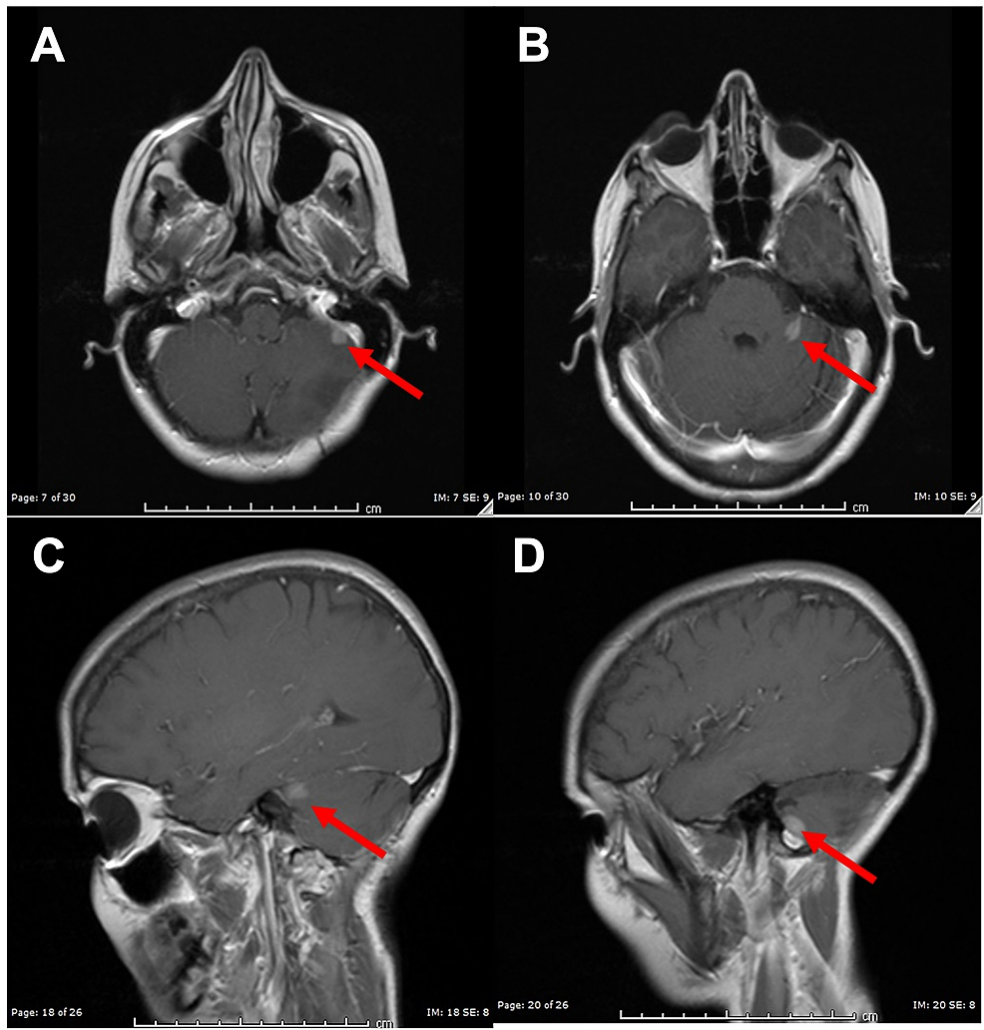
MR Images Following First Dose of Stereotactic Radiosurgery (A, B) Axial T2-weighted MR images demonstrating new enhancing lesions within the left cerebellum and resolution of the original mass after resection (red arrow). (C, D) Sagittal T1-weighted MR images with gadolinium contrast demonstrating new enhancing lesions within the left cerebellum and resolution of the original mass after resection (red arrow).

She underwent gross total resection, and the pathology showed the tumor to be metastatic breast carcinoma ER/PR-, HER-2+, suggesting that the tumor was metastatic from her previous breast primary tumor. She received 500 cGy over five fractions for a total of 2,500 cGy to the resection cavity and was restarted on infusional trastuzumab a month after completion of stereotactic radiosurgery (SRS). Six months later, a follow-up MRI showed new enhancing lesions in the anterior aspect of the left cerebellum measuring 1.2 x 0.7 cm and 0.8 x 0.7 cm (Figures 2A-2D).

**Figure 3:**
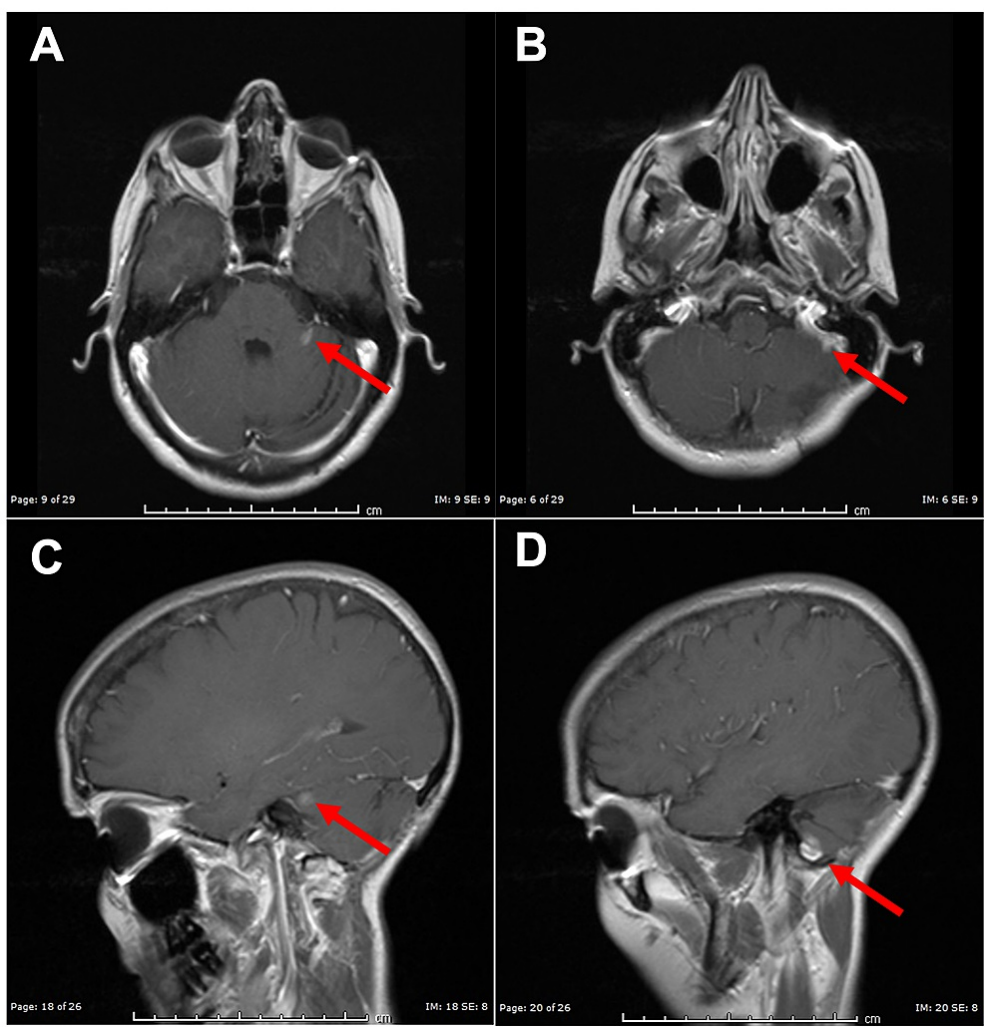
MR Images Following Second Dose of Stereotactic Radiosurgery (A, B) Axial T2-weighted MR images demonstrating improvement in two enhancing lesions within the left cerebellum after SRS. (C, D) Sagittal T1-weighted MR images with gadolinium contrast demonstrating improvement in two enhancing lesions within the left cerebellum after SRS. SRS: stereotactic radiosurgery.

At this time, she remained asymptomatic. The patient once again underwent SRS with 20 Gy delivered to the periphery of both lesions. MRI after six weeks showed a reduction of the lesions to 7 mm and 4 mm (Figures 3A-3D).

**Figure 4:**
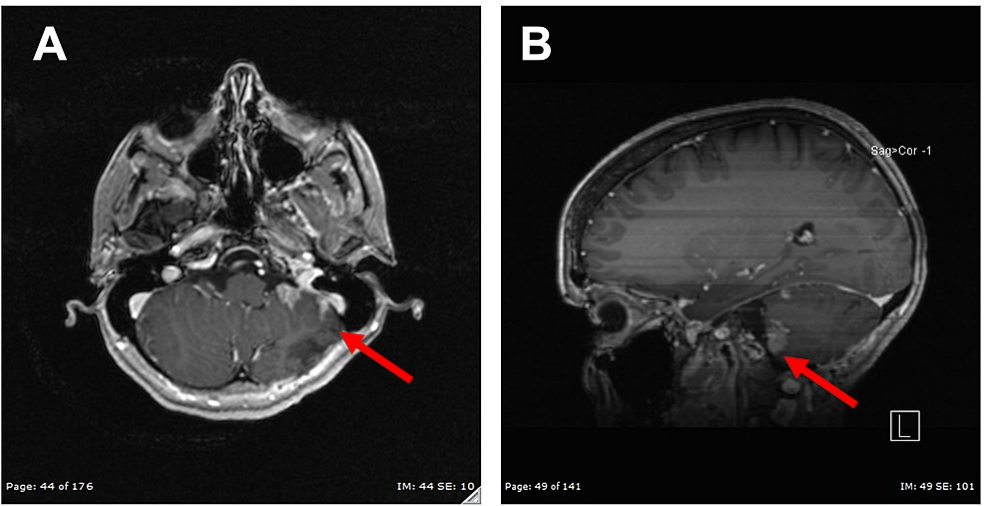
MR Images Showing Growth of Two Previous Lesions (A) Axial T2-weighted MR images demonstrating growth of two previously noted lesions as well as appreciation of new lesion within the superior anterior aspect of the left cerebellum (red arrow). (B) Sagittal T1-weighted MR images with gadolinium contrast demonstrating growth of two previously noted lesions as well as appreciation of new lesion within the superior anterior aspect of the left cerebellum (red arrow).

Four months after SRS to the two lesions, a follow-up MRI showed resolution of the larger nodule. The smaller lesion grew to approximately 8 x 4 x 5 mm and was abutting the left sigmoid sinus along with a new 3 x 2 x 1 cm mass thought to be residual from an older lesion. The decision at this time was to continue observation as she was asymptomatic, and the lesions were relatively small. At three months, a repeat MRI showed a new area measuring 1.0 x 0.2 x 0.5 cm along with the growth of the two previous lesions to 1.1 x 1.2 x 1.2 cm and 0.9 x 0.4 x 0.6 cm, none of which could be biopsied due to size of the lesions (Figures 4A, 4B).

**Figure 5:**
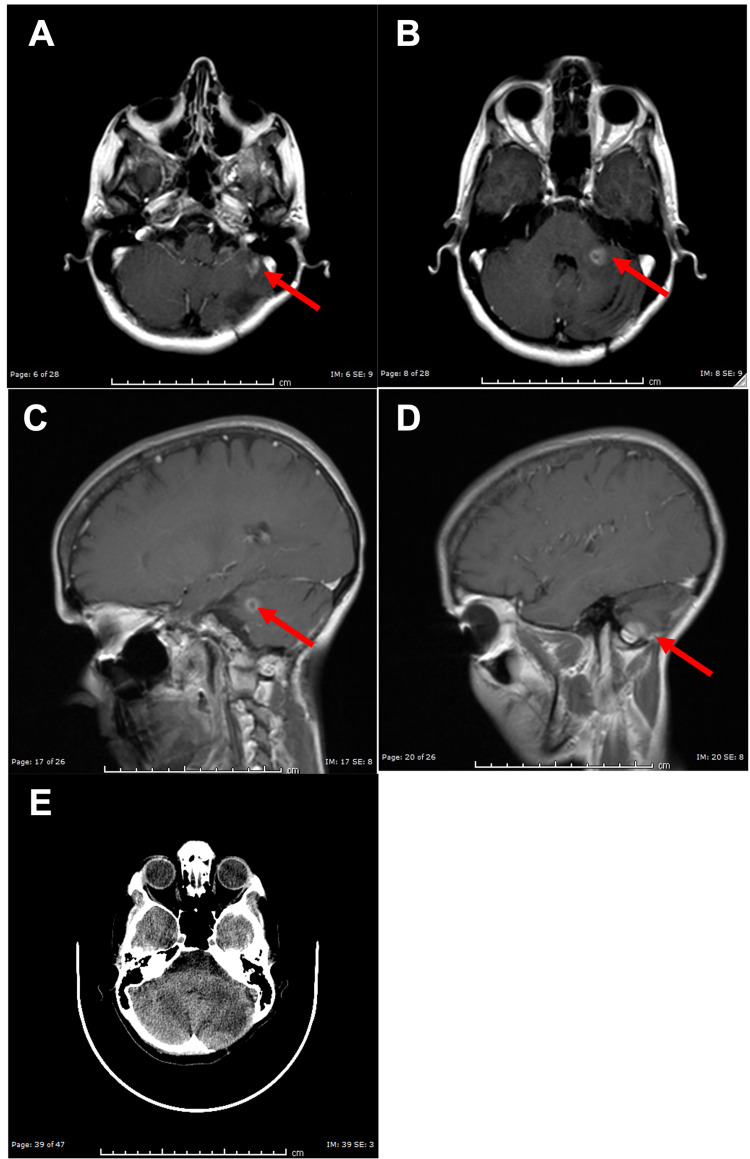
MR Images Following Final Dose of Stereotactic Radiosurgery (A, B) Axial T2-weighted MR images demonstrating new lesion within the brachium pontis (red arrow). (C, D) Sagittal T1-weighted MR images with gadolinium contrast demonstrating new lesion within the brachium pontis (red arrow). (E) (18F-FDG) PET with no abnormal uptake in the left cerebellum indicative of radiation necrosis. 18F-FDG: 2-deoxy-2-[fluorine-18] fluoro-D-glucose, PET: positron emission tomography.

At this time, infusional trastuzumab treatment was held and she underwent another round of SRS. Twenty Gray was administered to the periphery of the new lesion, and 16 Gy was administered to the periphery of the two lesions previously radiated. She was started on lapatinib, which is known to have central nervous system (CNS) penetration, along with capecitabine and dexamethasone. Repeat MRI showed stable lesions with new edema and a new lesion in the left middle cerebellar peduncle. A 2-deoxy-2-[fluorine-18] fluoro-D-glucose (^18^F-FDG) PET scan showed no abnormal uptake in the cerebellum suggestive of radiation necrosis, while MR spectroscopy suggested that the left middle cerebellar peduncle lesion was recurrent disease (Figures 5A-5E). These lesions have remained stable since this treatment was initiated.

**Figure 6:**
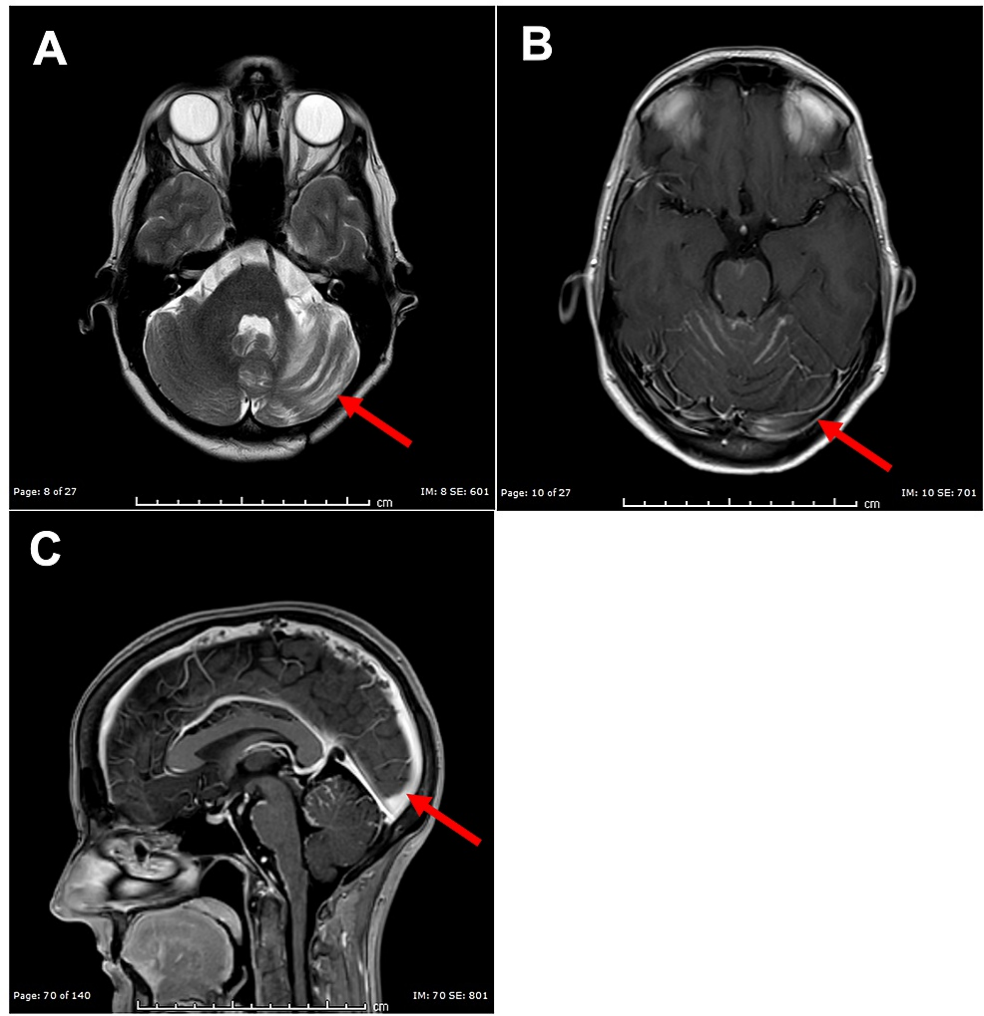
MR Images of Leptomeningeal Carcinomatosis (A) Axial T1 MR image with gadolinium contrast demonstrating onset of leptomeningeal carcinomatosis with stable cerebellar lesions (red arrow). (B) Axial T2 MR image demonstrating onset of leptomeningeal carcinomatosis with stable cerebellar lesions (red arrow). (C) Sagittal T1 MR image with gadolinium contrast demonstrating onset of leptomeningeal carcinomatosis with stable cerebellar lesions (red arrow).

Twenty-eight months after treatment of left cerebellar lesions, MRI showed leptomeningeal carcinomatosis (LC) bilaterally along the cerebellar folia and bilateral temporal lobes (Figures 6A-6C).

**Figure 7:**
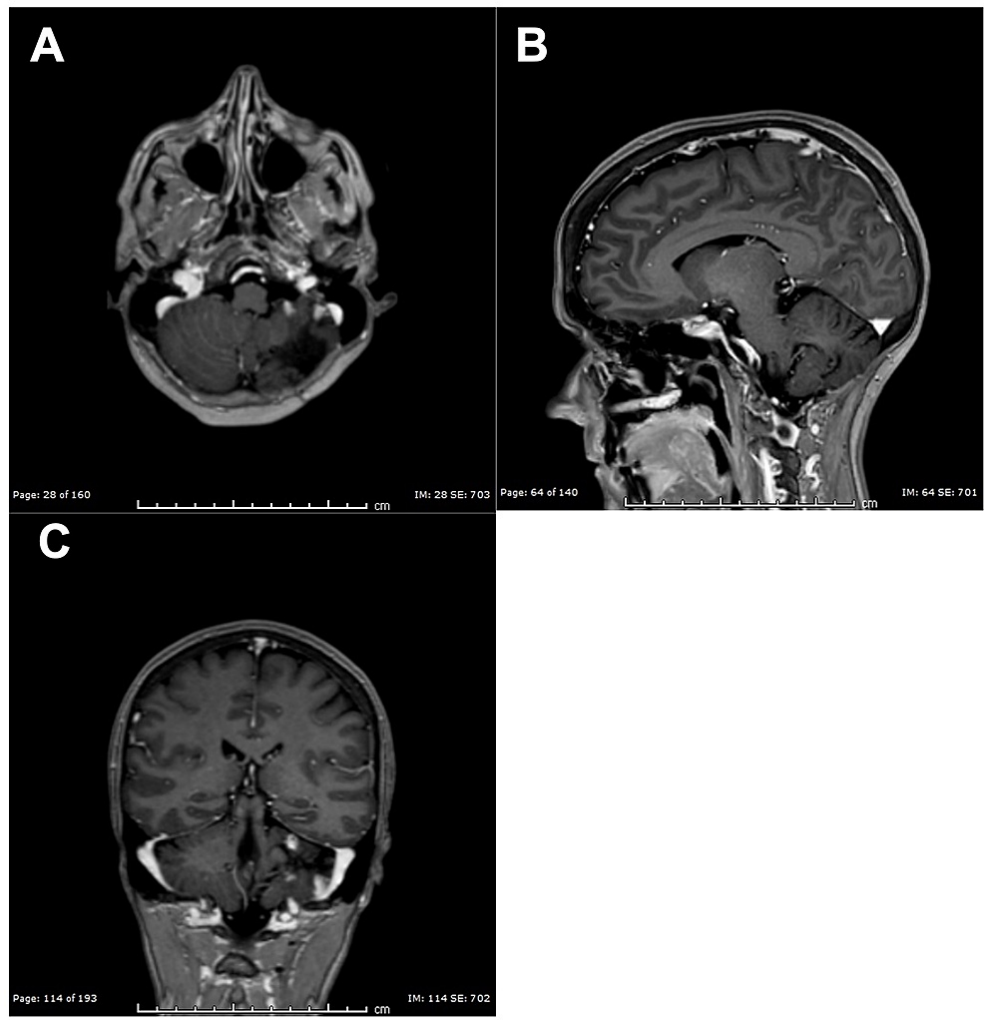
MR Images of Resolution of Leptomeningeal Carcinomatosis (A) Axial T2 MR image demonstrating resolution of leptomeningeal carcinomatosis with stable cerebellar lesions. (B) Sagittal T1 MR image with gadolinium contrast demonstrating resolution of leptomeningeal carcinomatosis with stable cerebellar lesions. (C) Coronal T1 MR image with gadolinium contrast demonstrating resolution of leptomeningeal carcinomatosis with stable cerebellar lesions.

Her physical exam and symptoms at this time were negligible. Lumbar puncture showed malignant cells in her cerebrospinal fluid (CSF). Lapatinib and capecitabine treatments were stopped, and intravenous fam-trastuzumab deruxtecan-nxki and intrathecal (IT) trastuzumab were started as both have CNS penetration. She began undergoing weekly lumbar punctures (LPs). After five weeks of therapy, LPs showed that her CSF was clear of malignant cells. After six months of therapy, her MRI showed complete response and resolution of LC, and the clinical exam remained unremarkable (Figures 7A-7C).

## Discussion

Metastatic brain cancer is a common and significant source of cancer-related death in the United States [6]. While larger and more symptomatic tumors are usually recommended for resection, smaller and asymptomatic masses can respond well to SRS alone. The tumors that are selected for SRS often recur locally and have an increased risk for radiation necrosis (RN) [7]. 

In the present case, one of the difficulties involved determining the etiology of small lesions after resection and SRS to the resection cavity. RN is a common occurrence after SRS, but due to the distance of the lesions from the radiation site, we considered recurrent metastases as a possibility in addition to RN. Understanding and differentiating between RN and recurrence are crucial to the treatment plan and outlook for patients. There are several modalities that can be utilized for doing so.

First, intravoxel incoherent motion MRI has been shown to be a reliable biomarker for establishing a lesion containing recurrent tumor or RN [8]. The advantage of this method is that it requires no intravenous contrast and a simpler modeling system than compared to other similar perfusion studies [9]. PET studies utilizing isotopically labeled amino acids have shown significant utility in discriminating RN from recurrence in the brain. PET positivity of O-(2-[^18^F]fluoroethyl)-L-tyrosine (^18^F-FET) and 6-[^18^F]-fluoro-L-3,4-dihydroxyphenylalanine (^18^F-FDOPA) has been shown to strongly correlate to higher tumor content within a lesion, while PET negativity corresponds to RN [10]. Both studies have been shown to have similar accuracy when compared against each other [11].

Finally, liquid biopsy has been recently shown to be efficacious in determining RN from malignancy. The HLA-DR-Vnn2 (human leukocyte antigen-DR isotype-vascular noninflammatory molecule 2) Index (DVI) can determine the content of a lesion simply by drawing blood. The average DVI corresponds strongly to a biopsy-confirmed diagnosis of RN or metastatic disease and thus should be considered when making treatment decisions [12]. 

The patient we present underwent two metabolic studies to determine the etiology of the lesions that appeared after SRS. The [^18^F]FDG PET scan showed no increased uptake in the left cerebellum indicating that the lesions were most likely RN. MRI spectroscopy showed slightly increased choline/creatinine ratio evidence of recurrence within the lesions. Dynamic susceptibility contrast perfusion imaging showed minimally increased perfusion, and 2-hydroxyglutarate lesion quantification was nondiagnostic. These conflicting studies made it difficult to move forward with a treatment plan. Ultimately, the decision was to proceed with the approved third-line treatment of the lesions with lapatinib, capecitabine, and high-dose corticosteroids [13]. This treatment plan was chosen since, if the lesions were malignant, the lesions would respond to the chemotherapeutics lapatinib and capecitabine. The dexamethasone would decrease any present edema and could slow necrosis if the lesions were indeed RN.

Treatment of LC varies as there is no consensus on treatment guidelines. First-line treatment of LC consists of methotrexate, liposomal cytarabine, and thiotepa, while HER-2 overexpressing (OE) metastases open the door for use of targeted therapies [14]. The use of trastuzumab delivered intrathecally (IT) has recently been studied for the treatment of LC in HER-2 OE leptomeningeal disease. 

An initial study in 2018 demonstrated that IT trastuzumab was beneficial in 13 patients with HER-2 OE leptomeningeal disease. All treatments were well tolerated and recommended for patients with HER-2 OE leptomeningeal disease [15]. A much larger study with 34 patients in 2019 demonstrated partial response or stable disease in 70% of participants. At six and 12 months, 24% and 12% of the cohort had progression-free survival with 56% and 41% overall survival, respectively [5]. Additionally, IT trastuzumab was compared to conventional IT chemotherapy and whole brain radiotherapy (WBRT) in leptomeningeal disease. Eighteen participants in the IT trastuzumab arm of this study demonstrated significant increases in craniospinal progression-free survival and overall survival [16].

In our patient, a previous report of successful IT trastuzumab use was used to determine dosing. This study demonstrated that 150 mg at a three-week interval was effective at maintaining sufficient levels of trastuzumab in serum and cerebrospinal fluid (CSF) [17]. Our patient was initiated on 100 mg every week. After 10 weeks, when CSF cytology demonstrated no malignant cells, the interval was increased to every three weeks at 100 mg until MRI showed resolution of LC.

To monitor response to therapy, CSF cytology was performed each week or every other week. Additionally, two- and six-month follow-up MRIs were performed to demonstrate response to therapy and resolution of LC. Other studies have demonstrated that CSF circulating tumor cells (CTCs) may be a more sensitive and efficacious method of monitoring treatment response and should be considered in patients with similar disease [18].

## Conclusions

There are currently no consensuses on treatment guidelines for treating leptomeningeal carcinomatosis. Here, we demonstrate successful treatment of leptomeningeal carcinomatosis from an ER/PR-, HER2+ breast carcinoma with intrathecal trastuzumab. Finally, we provide a complete literature review for the treatment of future patients with similar disease and the distinction of radiation necrosis from tumor recurrence.
